# *VMD2* mutational analysis in a Japanese family with Best macular dystrophy

**DOI:** 10.4103/0974-620X.57317

**Published:** 2009

**Authors:** Satomi Shiose, Shigeo Yoshida, Keijiro Ishikawa, Tatsuro Ishibashi

**Affiliations:** Department of Ophthalmology, Graduate School of Medical Sciences, Kyushu University, 3-1-1 Maidashi, Higashi-ku, Fukuoka 812-8582, Japan

Best macular dystrophy (BMD) is an early-onset, autosomal dominantly inherited disorder characterized by an egg yolk like yellowish macular lesion.[1] The appearance of the fundus of patients with BMD varies depending on the stage of disease and may be almost normal in the early stages.[2] Abnormal electrooculography (EOG) is considered a useful indicator of the disease and is used in its diagnosis. It was recently reported that mutations of the *VMD2* gene is responsible for BMD.[3] *VMD2* is an RPE-specific gene encoding bestrophin, a protein of unknown function. The onset and penetrance of the *VMD2* gene is varied, and several cases of BMD with different degrees of severity have been reported mainly in the Western world.

**Figure 1:**
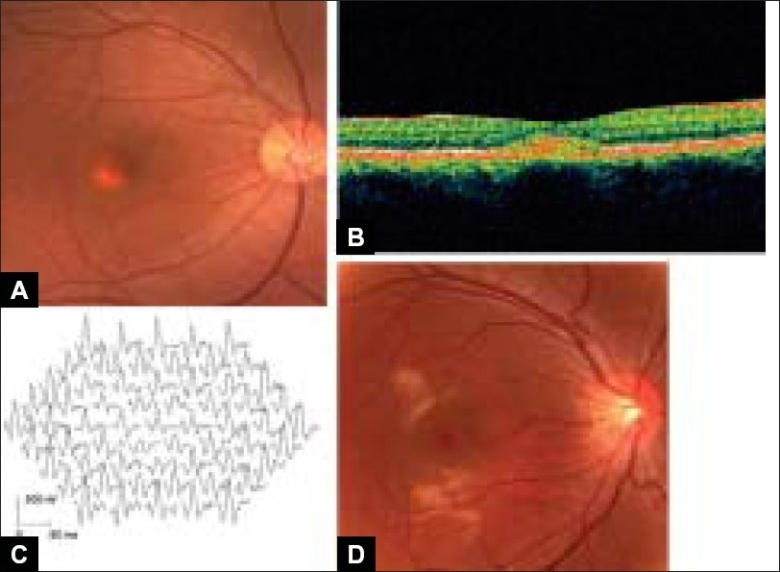
Fundus photograph, optical coherence tomograms (OCT) and multifocal ERGs of patient with Best macular dystrophy and her normal son (A) Fundus photograph of the right eye of the proband. The fundus shows yellowish material in vitelliform cysts in the macula, (B) OCT image through the yellowish lesion of the proband shows a highly reflective spindle-shaped zone between the photoreceptor layer and retinal pigment epithelial layer, (C) Multifocal ERGs of the right eye of the patient showing reduced responses from the macular area compared with those in a normal control, (D) Fundus photograph of the right eye of a 12-year-old son. The fundus shows irregular reflex in the macula

A 37-year-old Japanese woman was seen at Kyushu University complaining of blurred vision in her left eye. No member of her family had previously presented with or had symptoms of BMD. Her best-corrected visual acuity was 20/20 OD and 20/25 OS, and the intraocular pressures and anterior segments were normal. Fundus examination revealed typical scrambled egg-like lesions in the macula of both eyes [Figure 1A]. Optical coherence tomography (OCT; Carl Zeiss, Germany) of the yellowish lesion revealed a highly reflective, spindle-shape zone [Figure 1B]. Fluorescein angiography demonstrated hyper-fluorescence due to a window defect from the RPE atrophy, and hypo-fluorescence corresponding to the egg-like deposit from the early stage. Visual field, color vision tests, cone and rod ERGs were normal, but the multifocal ERGs were reduced in the macular area compared with those in a normal control [Figure 1C]. The light rise in the EOG was reduced (Arden ratio was 1.5 in the right eye and 1.3 in the left eye). Based on the clinical findings a diagnosis of BMD was made.

These clinical findings led us to recommend that other family members undergo ocular examinations. Fundus examination of the patient′s 12-year-old asymptomatic son, whose visual acuity was 20/20 OU, was almost normal showing only bilateral irregular reflex in the macula [Figure 1D]. Although we tentatively diagnosed the son as not having BMD, we could not completely exclude the possibility that he was an asymptomatic or preclinical carrier because of the variable expressivity of BMD. Because of her son′s lack of symptoms and because of the time-consuming nature of the examinations, the mother declined to let her son undergo any further ocular examinations including the EOG. However, she did agree to blood being taken both from herself and her son for molecular genetic analysis.

**Figure 2:**
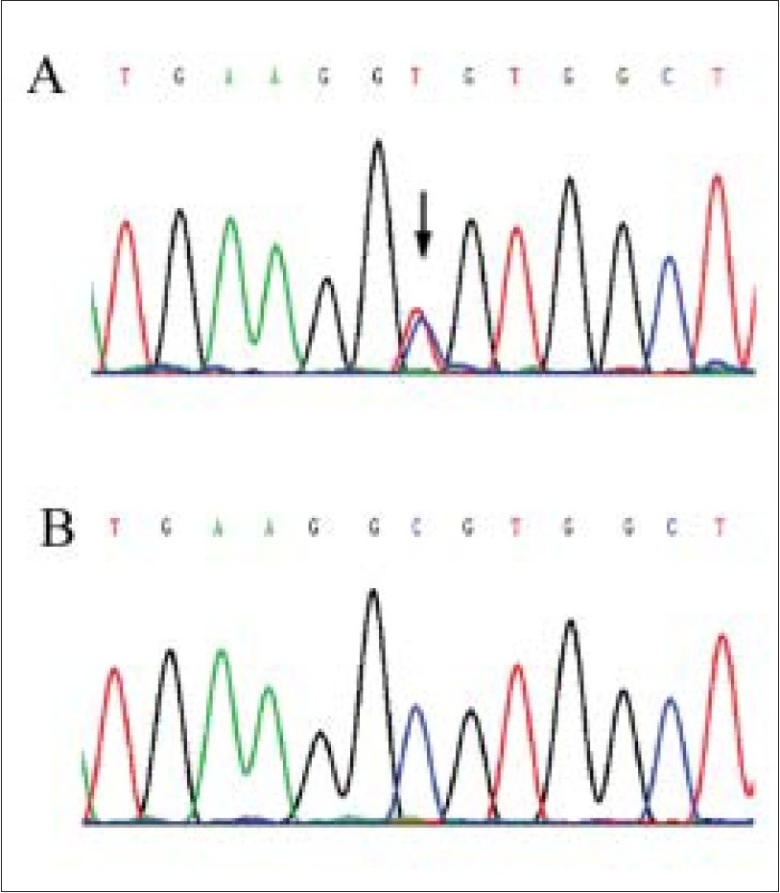
Sequence analysis of *VMD2* (A) The arrow indicates the heterozygous mutation of C to T change at nucleotide 584 (Ala195Val), (B) No equivalent mutation is detected in the subject's son or control subjects

After obtaining informed consent, blood was drawn, and direct sequencing[4] of all coding regions of the *VMD2* gene revealed a mutation causing a C to T change at nucleotide 584 (Ala195Val) in the mother but not in her son [Figure 2]. This mutation has been reported in a Caucasian[5] in the *VMD2* mutation database (http://www.uni-wuerzburg.de/humangenetics/vmd2.html). None of the other sequence alterations was detected in any of the 64 healthy, unrelated individuals without eye disease used as control subjects. Based on the molecular diagnosis, we were able to inform the mother that the possibility of her son inheriting the disease-causing mutation was very low.

Our case demonstrates the usefulness of the web-based database of mutations in *VMD2* for obtaining disease-causing sequence alterations. Increasing numbers of *VMD2* mutations are being posted on the web-based database, and it has become easier to identify mutations responsible for BMD. The fact that the same mutation found in the mother had been reported in a Caucasian suggests that the mutation is present in different ethnic groups.

Our study also demonstrated the value of molecular analysis of the *VMD2* gene, which can lead to a rapid and accurate diagnosis and the exclusion of BMD. It is a viable alternative to EOG for the diagnosis of BMD, which is currently accepted as the test for an early diagnosis of BMD. However, EOG is difficult to perform in young children, and in clinics where EOG is not available, blood samples or buccal swabs can be sent to a clinic where genetic analyses can be performed.
